# Improved Tetanic Force and Mitochondrial Calcium Homeostasis by Astaxanthin Treatment in Mouse Skeletal Muscle

**DOI:** 10.3390/antiox9020098

**Published:** 2020-01-23

**Authors:** Mónika Sztretye, Zoltán Singlár, László Szabó, Ágnes Angyal, Norbert Balogh, Faranak Vakilzadeh, Péter Szentesi, Beatrix Dienes, László Csernoch

**Affiliations:** 1 Department of Physiology, Faculty of Medicine, University of Debrecen, 4032 Debrecen, Hungary; sztretye.monika@med.unideb.hu (M.S.); singlar.zoltan@med.unideb.hu (Z.S.); laszlo.szabo@med.unideb.hu (L.S.); angyal.agnes@med.unideb.hu (Á.A.); balogh.norbert@med.unideb.hu (N.B.); faranak.vakilzadian@med.unideb.hu (F.V.); szentesi.peter@med.unideb.hu (P.S.); dienes.beatrix@med.unideb.hu (B.D.); 2 Doctoral School of Molecular Medicine, University of Debrecen, 4032 Debrecen, Hungary

**Keywords:** skeletal muscle, intracellular calcium, mitochondrial calcium, excitation contraction coupling, force, astaxanthin, retinol

## Abstract

Background: Astaxanthin (AX) a marine carotenoid is a powerful natural antioxidant which protects against oxidative stress and improves muscle performance. Retinol and its derivatives were described to affect lipid and energy metabolism. Up to date, the effects of AX and retinol on excitation-contraction coupling (ECC) in skeletal muscle are poorly described. Methods: 18 C57Bl6 mice were divided into two groups: Control and AX supplemented in rodent chow for 4 weeks (AstaReal A1010). In vivo and in vitro force and intracellular calcium homeostasis was studied. In some experiments acute treatment with retinol was employed. Results: The voltage activation of calcium transients (V_50_) were investigated in single flexor digitorum brevis isolated fibers under patch clamp and no significant changes were found following AX supplementation. Retinol shifted V_50_ towards more positive values and decreased the peak F/F_0_ of the calcium transients. The amplitude of tetani in the extensor digitorum longus was significantly higher in AX than in control group. Lastly, the mitochondrial calcium uptake was found to be less prominent in AX. Conclusion: AX supplementation increases in vitro tetanic force without affecting ECC and exerts a protecting effect on the mitochondria. Retinol treatment has an inhibitory effect on ECC in skeletal muscle.

## 1. Introduction

The energy required for muscle contraction arises from the breakdown of ATP. The mitochondria being the site of most ATP production play a critical role in skeletal muscle function [1]. Oxidative phosphorylation, the aerobic production of ATP highly depends on the morphological and functional state of mitochondria, including appropriate intra-mitochondrial calcium levels. In addition to ATP synthesis, mitochondria are also known to be both the major source and the target of reactive oxygen species (ROS) at the same time. Under physiological conditions the production of ROS, which are crucial in signaling processes, and the activity of the antioxidant systems are strictly balanced. The latter is responsible for the removal of ROS and comprised of exogenous and endogenous antioxidant molecules and enzymes as catalase, superoxide dismutase (SOD), lacto-peroxidases, and glutathione peroxidase. Skeletal muscles generate remarkable amounts of ROS even during inactivity [2,3]. The majority of them are produced in the mitochondria [4]. During moderate exercise the production of free radicals increases, the up-regulation of antioxidant occurs via redox signaling and the oxidant-antioxidant balance is restored [5]. On the other hand, during physical training the redox balance is disturbed, and this unbalance has been shown to decrease muscle performance due to the oxidative injury and muscle fatigue evoked by ROS [6,7]. The elevated ROS level has also been found to contribute to muscular damage [8].

Much attention has been dedicated to the supplementation of various types of food-derived antioxidants as potential agents in the prevention of oxidative stress-related processes, especially resulting in a greater force production and fatigue resistance in endurance trainings, as well as providing protection against muscle fatigue [9,10,11,12]. The protective function of the well-known exogenous antioxidants, vitamin A, C, and E which can be taken with food or with supplementation, have already been demonstrated in several aspects of skeletal muscle physiology: e.g., force production, glucose uptake, insulin sensitivity, ion pump functions, and mitochondrial biogenesis [4]. Effects of vitamin C and E are well documented, however, there are some controversial results when these antioxidant agents were not effective, or they even increased the incidence of human diseases [1,13,14,15,16]. Vitamin A (retinol) is also involved in redox processes and considered as potential antioxidant in exercise-induced oxidative stress however, in certain studies its effects were not beneficial, as in skeletal muscle it increased oxidative stress resulting in tissue damage [17,18]. Retinol and its derivatives are commonly referred to as retinoids, irrespectively of their biological activity. Retinoids are present in circulation at high concentrations as retinol bound to retinol-binding protein and are then taken up by cells for storage or converted into retinoic acid (Rac) which regulates several retinoid-responsive genes. Rac can bind to and activate nuclear hormone receptors and has notable effects on lipid and energy metabolism and was described to improve muscle performance in mice by increasing glucose uptake [19,20].

Promising results have been obtained during the administration of the fat soluble red-colored pigment astaxanthin (AX), which belongs to the xanthophyll subgroup of marine carotenoids. Nishida and coworkers found that AX presents the strongest capacity to quench singlet oxygen when compared to other antioxidants [21]. Sawaki and colleagues showed that a dose of 6 mg AX per day for four weeks resulted in decreased levels of lactic acid during a 1200-m sprint [22]. A double-blind study on humans revealed that natural AX increases fat utilization during exercise [23]. Wolf and coworkers showed that AX stimulates mitochondrial respiration by maintaining a higher membrane diffusion rate, allowing increased oxygen uptake, and improves energy availability to muscles [24]. Carotenoids, precursors of vitamin A, are natural pigments found in many fruits and legumes and are known to have a very similar chemical structure to retinol [25]. Unlike beta-carotene, AX is able to readily cross the blood-brain barrier, however it cannot be converted to retinol and thus to vitamin A and therefore cannot support retinol-specific processes such as vision [26]. AX is extensively produced by krill, arctic shrimp, and algal species such as *Haematococcus pluvialis* and also by the yeast *Phaffia rhodozyma* [27]. A recent review summarizes the beneficial effect of this special fat soluble carotenoid with strong antioxidant capacity, including its impact on muscle physiology [28]. There are cumulating data suggesting that AX can reduce oxidative stress and prevent against mitochondrial damage by permeating through mitochondrial membranes, which is a unique feature among antioxidants [29,30].

Excitation-contraction coupling (ECC) is the series of well-coordinated steps whereby an action potential triggers a cytoplasmic calcium signal that initiates the contraction of muscle [31]. ECC occurs in the triad, a distinctive structure formed by the interface between the T-tubule and the terminal cisternae of the sarcoplasmic reticulum (SR). Activation of the dihydropyridine receptor (DHPR) in the T-tubule membrane opens the ryanodine receptor (RyR) in the SR membrane and initiates the release of calcium from the SR into the intracellular space [28,32,33,34]. This calcium is a key regulator of a variety of cellular processes, including muscle contraction and mitochondrial function. Mitochondria actively contribute to the spatio–temporal distribution of intracellular calcium concentrations [35,36]. Any perturbation in this precisely orchestrated machinery may end in muscle dysfunction and/or reduced muscle performance. ROS appears to target multiple sites of the ECC: It has been shown to affect DHPRs, and RyRs as well, indicating that the cellular redox state is a dominant factor in calcium homeostasis, which is able to affect muscle contraction. Subsequently, or independently, mitochondrial calcium uptake is also affected by ROS [37,38,39,40,41]. How effective exogenous antioxidants are against ROS-induced damages in ECC machinery and/or mitochondrial calcium handling is still elusive.

In this work we describe the changes of muscle force, ECC and mitochondrial calcium homeostasis in AX fed mice. Since AX is suggested to be used as a dietary supplement for healthy individuals, we aimed to describe its effects under physiological conditions on healthy muscles. We also examined the effect of retinol—a structurally similar compound to AX—on the ECC mechanism and fatigability of fast twitch skeletal muscles and propose a model of AX actions. Apart from the metabolic aspects, which are only indirectly addressed here, our results help to understand the physiological consequences of antioxidant administration on healthy people. Our results support the beneficial effects of AX in mammals.

## 2. Materials and Methods

### 2.1. Animal Care

Animal experiments conformed to the guidelines of the European Community (86/609/EEC). The institutional Animal Care Committee of the University of Debrecen approved the experimental protocol (3-1/2019/DEMAB). The mice were housed in mesh covered plastic cages and were kept at normal room temperature (22–25 °C). They had access to water ad libitum and were fed with pelleted mouse chow. Room illumination was on automated cycle of 12 h dark and 12 h light. 

### 2.2. Astaxanthin (AX) Diet

In our experiments we used AstaReal A1010, an astaxanthin-rich natural product, composed from crushed and spray-dried aplano-spores of the microalgae *Haematococcus pluvialis.* AstaReal A1010, a pasteurized algal meal containing 5–5.6% esterified AX is presented as a water insoluble red powder with characteristic algal odor. Eighteen C57Bl6 male 4–6 months old male mice were separated randomly into two groups. AX diet (per os) lasted for 4 weeks (AX group) while littermates were fed with standard rodent chow (CTRL group). The special chow was prepared with the addition of 4 g/kg of AstaReal A1010 (dissolved in 100% ethanol) to the standard rodent pellet for a final concentration of 0.02% AX. This concentration was chosen according to the literature (see Aoi et al., 2003; 2018) [42,43]. AX from *Haematococcus pluvialis* algae has been reviewed by the US FDA and cleared for marketing as a new dietary ingredient. It has been used safely by itself for over 20 years now in doses of 4 to 40 mg daily for up to 12 weeks, or 12 mg daily for 6 months [44].

### 2.3. In Vivo Experiments

#### 2.3.1. Body Weight Measurement

The body weight was measured at the beginning (day 1) and after the four weeks feeding period (day 28) for each individual mouse in both control and AX group. The body weight increase was averaged by groups.

#### 2.3.2. Forepaw Grip Test

The force of forepaw was measured similarly as described in our earlier report [45]. In short, once the animals reliably grasped the bar of the grip test meter, it was then gently pulled away from the device holding its tail. The maximal grip force was reached when the animal let off the bar and it was stored by an online connected computer. The test was repeated 10–15 times to obtain a single data point. The grip force was normalized to the body weight of each animals. Measurements were carried out before and after the four weeks AX feeding period.

### 2.4. In Vitro Experiments

Animals were anaesthetized and sacrificed following an approved protocol (3-1/2019/DE MAB). After CO_2_ overdose and manual cervical dislocation, the m. flexor digitorum brevis (FDB) and the m. extensor digitorum longus (EDL) from the hind limb were dissected manually.

#### 2.4.1. Measurement of EDL Muscle Force

Muscle contractions were measured as described previously [46]. Briefly, EDL muscles were placed horizontally in an experimental chamber. They were continuously superfused (10 mL/min) with Krebs’ solution which was equilibrated with carbogen (95% O_2_ and 5% CO_2_) at room temperature. One end of the muscle was attached to a capacitive mechano-electric force transducer (Experimetria, Budapest, Hungary) while the other to a rod. Muscles were then stretched to a length that delivered the maximal force response followed by a 5 min interval when they were allowed to equilibrate. Two platinum electrodes touching the muscle were used to deliver supramaximal pulses (5 V) of 2 ms in duration. These pulses with 1 Hz frequency were used to elicit at least 10 single twitches which were measured under these conditions from every muscle. To develop a tetanus, single pulses (5 V, 2 ms) were applied with 200 Hz frequency for 200 ms. Force responses were digitized at 2 kHz using TL-1 DMA interface and stored on an online connected computer with Axotape software (Axon Instruments, Foster City, CA, USA). The force was finally normalized to the cross sectional area of each EDL muscle.

#### 2.4.2. Isolation of Single FDB Muscle Fibers

All calcium measurements were carried out on single skeletal muscle fibers from the FDB muscles of the mouse. The manual dissection of FDB muscle in normal Tyrode’s solution was followed by an enzymatic dissociation in minimal essential media containing 0.2% Type I collagenase (Sigma-Aldrich, St. Louis, MO, USA, cat. no. SCR103) at 37 °C for 50 min in a calcium free Tyrode’s solution. After the enzymatic treatment FDB muscles were placed in normal Tyrode’s solution and stored in the refrigerator at 4 °C until use for up to 36 h. Single FDB fibers were obtained after gently triturating the muscle with a pipette [47,48].

#### 2.4.3. Confocal Microscopy and Image Analysis

Images of rhod-2 fluorescence were acquired with a laser scanning confocal microscope (Zeiss 5 Live, Oberkochen, Germany) equipped with a 20× air objective which allowed excitation of rhod-2 at 543 nm, with emission collected above 550 nm with a long pass filter. Line-scan image recordings were synchronized with the application of the depolarizing pulses (100 or 200 ms long) via pClamp 11.0 (Molecular Devices, San Jose, CA, USA), see below. The time resolution was 0.5 ms per line whereas the spatial resolution was 0.24 µm/line. Baseline fluorescence (F_0_[x]) was calculated by averaging 15–20 lines in the time domain prior to the first depolarizing pulse. Fluorescence intensity was expressed as normalized to F_0_[x] (F[x]/F_0_[x]). Peak F/F_0_ values were then obtained by averaging the data points in the spatial domain and plotted at close to maximal depolarizations. When a series of tetanic depolarizing pulses were applied a single exponential function:

*y* = *y*_0_ + *a*e^−*bx*^ (1)

was used to fit the time course of the changes of the maximal F/F_0_.This then enabled the estimation of SR calcium content as described in our earlier report [49]. In the equation above *x* is the number of tetanic pulses applied, *b* is the time constant of SR calcium depletion, *a* is the remaining calcium in the SR following the last depolarizing pulse and *y*_0_ is the difference between the total and the remaining SR calcium content following the 8th tetanic pulse.

#### 2.4.4. Voltage Clamp

Isolated FDB fibers were placed in the external solution and voltage-clamped (Axoclamp 200B, Axon Instruments, Foster City, CA, USA). Changes in cytosolic calcium were recorded, following the application of depolarizing voltages and simultaneously imaged using a confocal microscope (Zeiss 5 Live, Oberkochen, Germany). Fibers were dialyzed through the patch pipette with the rhod-2 and 10 mM ethylene glycol-bis(2-aminoethylether)-N,N,N′,N′-tetraacetic acid (EGTA) containing internal solution and the application of depolarizing pulses was started 15–20 min following the seal formation to assure enough loading time. The ambient temperature was 20–22 °C. The holding potential was set to –80 mV and the pipette resistance varied between 2 and 4 MΩ. The linear capacitive currents were corrected by analog compensation. The Boltzmann function was used to describe the voltage (V_m_) dependence of activation:

Ca (V) = Ca_max_/(1 + exp(−(V_m_ − V_50_))/k) (2)

Here V_50_ is the transition voltage and 1/k the limiting logarithmic slope. Individual data points were normalized to Ca_max_ and plotted as a function of the membrane potential to display the voltage dependence of the activation.

In some experiments isolated FDB fibers were incubated with 10 µM retinol for 3 h in normal Tyrode’s at 4 °C.

#### 2.4.5. Measurement of Mitochondrial Calcium Uptake

The changes in mitochondrial calcium levels in single FDB fibers following repetitive stimulation were monitored with rhod-2 following Ainbinder and coworkers [50]. FDB fibers were loaded with 5 µM rhod-2-AM for 15 min at room temperature and then washed with a dye free normal Tyrode’s solution. Fibers were electrically stimulated (S88 Stimulator, Grass Technologies, Warwick, RI, USA) through a pair of platinum electrodes placed close to the fiber of interest. A single or a series of 5 consecutive tetani (500 ms duration, 100 Hz) were applied at supramaximal activating voltage for each cell. Time series x-y images (512 × 512 pixels, 0.5 ms/pixel) were taken at rest, following the 1st and 5th tetanus. Calculation of rhod-2 fluorescence values originating from the mitochondria (F_mito_) were performed the following way: a line was drawn parallel to the longitudinal axis of the fiber and the fluorescence was calculated at the peaks (I-band fluorescence, representing mitochondria (F_I-band_)) and at troughs (A-band, fluorescence, baseline, F_A-band_). The normalized mitochondrial calcium uptake expressed as F_mito_ was calculated as (F_I-band_ – F_A-band_)/F_A-band_.

### 2.5. Experimental Solutions

Kreb’s solution (in mM): 135 NaCl, 5 KCl, 2.5 CaCl_2_, 1 MgSO_4_, 10 HEPES, 10 glucose, 10 NaHCO_3_; pH 7.2.

Normal Tyrode’s solution (in mM): 137 NaCl, 5.4 KCl, 1.8 mM CaCl_2_, 0.5 MgCl_2_, 11.8 HEPES; 1 gL^-1^ glucose; pH 7.4.

Ca^2+^ free Tyrode’s solution: same as above except 5 mM EGTA was added and no CaCl_2_ was present.

External solution (in mM): 140 TEA-CH_3_SO_3_, 1 CaCl_2_, 3.5 MgCl_2_, 10 HEPES, 1 4-AP, 0.5 CdCl_2_, 0.3 LaCl_3_, 0.001 TTX (citrate), and 0.05 BTS (N-benzyl-p-toluene sulphonamide). pH was adjusted to 7.2 with TEA-OH and osmolality was adjusted to 320 mOsm with TEA methanesulfonate.

Internal solution (in mM): 110 N-methylglucamine, 110 L-glutamic acid, 10 EGTA, 10 Tris, 10 glucose, 5 Na_2_ ATP, 5 PC Tris, 0.5 rhod-2, 3.56 CaCl_2_, and 7.4 mM MgCl_2_ were added for a nominal 1 mM [Mg^2+^] and 100 nM [Ca^2+^]. pH was set to 7.2 with NaOH and osmolality to 320 mOsm with N-methylglucamine.

Retinol treatment: Retinol was prepared as a 10 mM stock in 100% EtOH.

Fluorescent dyes (rhod-2 tripotassium salt and AM) were purchased from Invitrogen (Thermo Fischer, Waltham, MA, USA). All other chemicals were purchased from Sigma-Aldrich (St. Louis, MO, USA). Astaxanthin (AstaReal A1010) was a kind gift for research purposes from AstaReal Sweden Co Ltd. (Nacka, Sweden) supplied in 10 g vacuum package in an airtight aluminum bag carefully protected from air, heat and light. During laboratory handling and AX supplemented chow preparation we maintained these precautions.

### 2.6. Statistical Analysis

Pooled data were expressed as mean ± standard error of the mean (SEM). In case of in vitro force measurement, the mean and SEM were calculated as the averages of the values of muscles from the same animal, while the number of samples was the number of animals in the given group. The differences between control mice and animals on AX diet were assessed using unpaired t-test extended with D’Agostino-Pearson normality test. To compare CTRL, AX and retinol data one way analysis of variance (ANOVA) and all pair wise Bonferroni’s multiple comparison method was carried out using the statistical program Prism (GraphPad Software, San Diego, CA, USA).

## 3. Results

### 3.1. Body Weight Gain Decreases, Grip Force Increases in AX Mice

**Figure 1:**
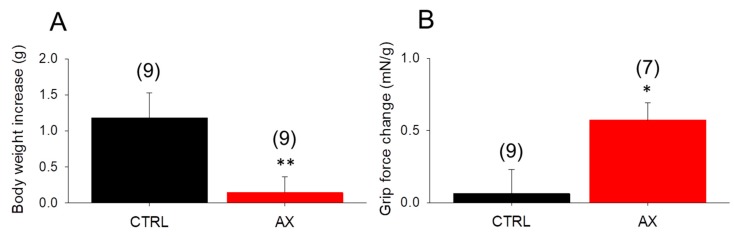
Significant in vivo changes following 4 weeks astaxanthin (AX) diet. (**A**) The body weight increase was less prominent in the AX group. (**B**) The increase of maximal grip force normalized to body weight was greater following AX diet. Numbers in parenthesis denote the number of animals. Averages of values were measured on the first and last day of AX diet. Significant differences between CTRL and AX were marked with * *p* < 0.05 and ** *p* < 0.01, respectively.

Since AX was proposed as a potent antioxidant affecting cell metabolism, the chronic effect of AX administration on body weight was evaluated. We measured the body weight of mice fed with standard rodent chow and AX supplemented chow, on day 1 and on day 28 of the feeding. The average body weight gain (from 27.7 ± 1.0 to 28.8 ± 0.8 and 28.1 ± 0.7 to 28.2 ± 0.7, in CTRL and AX, respectively) was found to be significantly smaller in AX mice than in CTRL animals (Figure 1A). This was not due to decreased food intake since the average intake was 0.21 ± 0.01 vs 0.22 ± 0.02 g/day/g body weight for CTRL and AX, respectively.

To check the in vivo muscle performance of the animals, grip tests were used also on the beginning and the end of the special diet period. AX supplemented mice showed an increased normalized grip force after 4 weeks (Figure 1B).

### 3.2. Tetanic Force Increases in AX Mice

**Figure 2:**
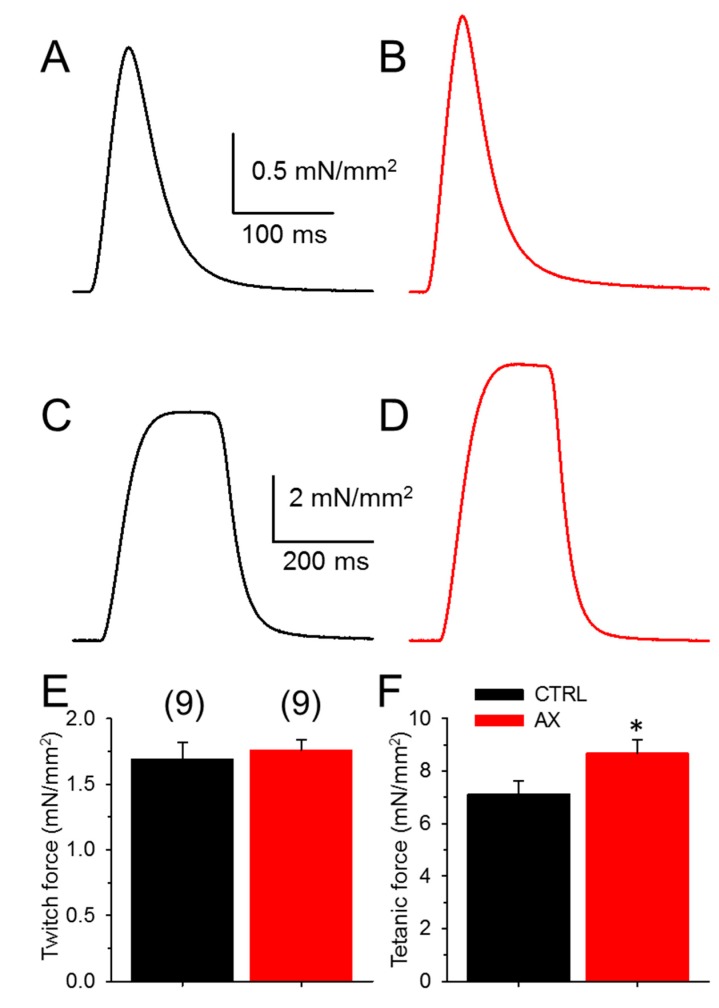
Increased tetanic force in EDL muscles after AX treatment. Representative ex vivo twitch (**A**,**B**) and tetanic (**C**,**D**) force on EDL muscle from CTRL (black) and AX fed (red) mice stimulated at 0.5 or 200 Hz, respectively at room temperature (22–24 °C). The force was normalized to the cross sectional area of the muscle. The numbers in parenthesis indicate the number of animals studied. The same numbers apply for both panels (**E**) and (**F**). * denotes significant difference between CTRL and AX, at *p* < 0.05.

In order to determine whether AX administration might alter muscle functions directly, in vitro muscle strength was investigated. There was no difference in the mean amplitude of the normalized single twitches of EDL muscles (Figure 2A,B,E) between AX supplemented and the control animals (Table 1). This is somewhat predictable as the cross sectional area of the EDL muscles was not different between the groups (1.30 ± 0.07 mm^2^ vs. 1.27 ± 0.05 mm^2^ for CTRL and AX, respectively). On the other hand, AX supplementation significantly increased tetanic force (Figure 2C,D,F). We did not find significant differences between the time to peak and half relaxation time of EDL muscles in CTRL and AX mice (Table 1).

### 3.3. SR Ca^2+^ Release and its Voltage-Dependence Remain Unaltered in AX Mice

**Figure 3:**
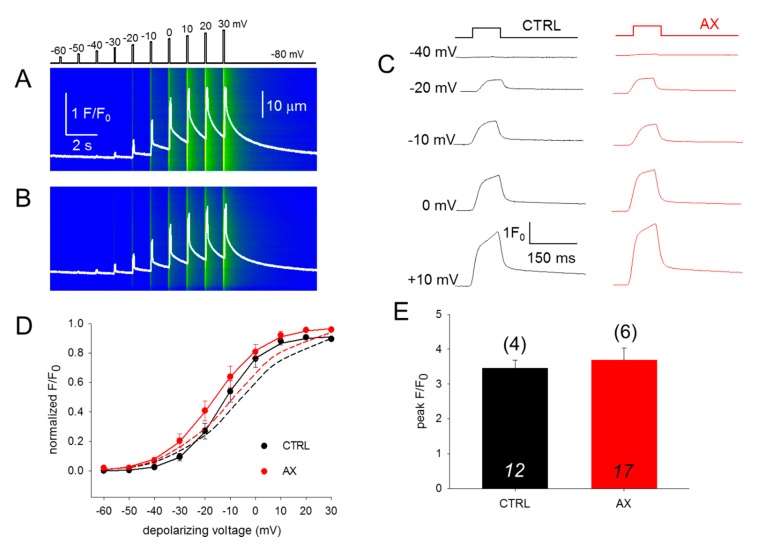
Unchanged release channel sensibility to voltage activation of the FBD muscles observed following 4 weeks AX diet. (**A**,**B**) Representative line-scan images of rhod-2 fluorescence normalized to the baseline value F_0_(x) in a control (**A**) and AX (**B**) cell subjected to a successive rectangular depolarizing voltage steps under whole-cell voltage-clamp. The cells were held at –80 mV and were perfused with an internal solution containing 10 mM EGTA. The calcium transients were elicited by 100 ms long progressively increasing membrane depolarizations ranging from –60 mV to +30 mV, with 10 mV increments. The delay between two pulses was 1100 ms. The white trace is the temporal profile of the normalized fluorescence obtained by averaging 50 lines in the spatial domain normalized to average resting F_0_(x) values. (**C**) The temporal profiles of the normalized fluorescence at individual 100 ms long depolarizing pulses as indicated for single FDB fibers from CTRL and AX mice. (**D**) Voltage dependence of the calcium transients. The normalized F/F_0_ values were fitted with a Boltzmann function (see Equation (2) in Methods) and then normalized to the obtained maximum for a given fiber and lastly averaged over the fibers in each group. The continuous lines represent the best fit of the Boltzmann function to the average values with the following parameters: V_50_ = –13.03 and –16.57 mV, k = 7.91 and 10.04 for CTRL (*n* = 12 fibers, *N* = 4 mice) and AX (*n* = 17 fibers, *N* = 6 mice), respectively. The dashed lines indicate the best fit of the Boltzmann function of the individual depolarizing pulses (applied every 2 min) with the following parameters: V_50_ = –6.73 and –9.18 mV, and k = 12.47 and 12.57 for CTRL and AX, respectively. (**E**) Pooled data for peak F/F_0_ values obtained at close to maximal depolarizing pulses (+10 mV). The numbers in italics indicate the number of cells studied, whereas the numbers in parenthesis denote the number of animals studied.

To assess the effect of AX treatment on the coupling between DHPR and RyR1, calcium transients were studied. We investigated 100-ms long rectangular depolarizations between –60 mV and +30 mV with 10 mV accrual. The whole-cell voltage-clamp technique was used to record calcium transients in single isolated FDB fibers in parallel with confocal microscopy. Figure 3A,B display two representative line-scan images from a CTRL and an AX fiber. Figure 3D compares the voltage dependence of the normalized fluorescence from 12 CTRL (black) and 17 AX fibers (red) obtained from independent experiments similar to those shown in Figure 3A,B. Neither in the voltages of half-maximal activation, nor in the slopes of the fitted curves were alterations found comparing data collected on CTRL fibers to that of AX fibers, reflecting identical Ca^2+^ release activation in the two samples. These results indicate that the AX treatment does not have an impact on the release channel activation and altogether the ECC machinery remains unaltered. Similar results were obtained with the application of single 100-ms-long gradually increasing membrane depolarizations (Figure 3C,D dashed lines). The mean value of the peak fluorescence at close to maximal activation (+10 mV) was not statistically different between the two groups (3.46 ± 0.22 vs. 3.68 ± 0.35 for CTRL and AX, respectively; Figure 3E). As the duration of the depolarizing pulse was always constant (100 ms) the areas of the F/F_0_ transients were calculated from the beginning till the end of the pulse and were found not to be statistically different (172.1 ± 17.9 ms for CTRL vs 176.4 ± 19.2 ms for AX, respectively; *p* > 0.8). These findings suggest that the greater tetanic force observed in AX mice is not the consequence of an increased calcium release.

### 3.4. Mitochondrial Calcium Uptake is Reduced in AX Mice

**Figure 4:**
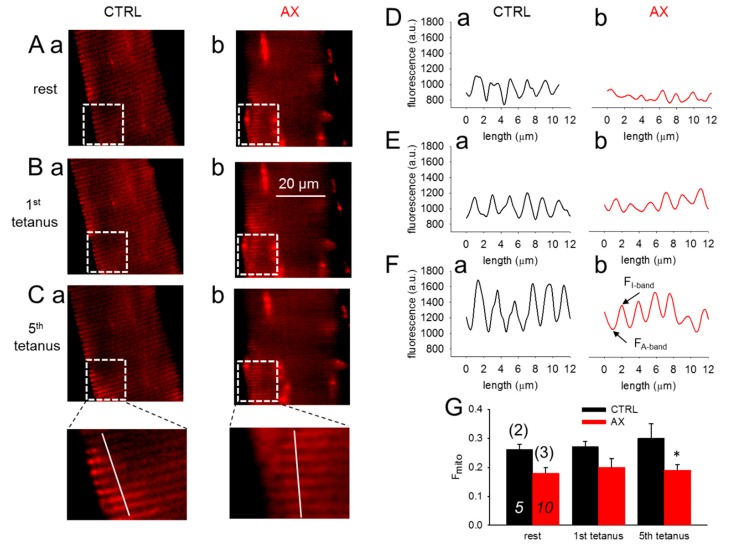
Decreased activity-dependent changes in mitochondrial calcium levels in single FDB fibers from AX mice. (**A**–**C**) Representative images of rhod-2 fluorescence in FDB fibers before and after tetanic stimulation (1st and 5th tetanus) in CTRL (**a**) and AX (**b**). (**D–F**) Representative mitochondrial fluorescence intensity profiles plotted from the highlighted square areas in images from Aa, Ba, Ca and Ab, Bb, Cb, respectively. (**G**) Average (±SEM) change of normalized mitochondrial fluorescence (F_mito_) calculated as (F_I-band_ – F_A-band_)/F_A-band_ in CTRL and AX FDB fibers at rest and following the tetanic stimulation. The numbers in italics indicate the number of cells studied, whereas the numbers in parenthesis denote the number of animals studied. Note the tendency of mitochondrial calcium accumulation and the differences of the normalized F_mito_ observed in control and AX. * denotes significant difference between CTRL and AX, at *p* < 0.05.

Figure 4A–C compares confocal line-scans of rhod-2 fluorescence in a CTRL and an AX FDB muscle fiber at rest (Figure 4A) and in response to one (Figure 4B) and five (Figure 4C) consecutive tetanic depolarizing pulses to supramaximal voltages applied via platinum electrodes placed adjacent to the cell of interest. The fluorescence averaged over the spatial domain illustrate that the rhod-2 fluorescence increases after tetanic stimulation (Figure 4D–F). The average fluorescence increases were found to be smaller in AX fibers (Figure 4G). The traces and the pooled data indicate the activity dependent mitochondrial calcium uptake. Note that less calcium is accumulated by the mitochondria in AX treated than in CTRL fibers which is likely to be the explanation for the greater tetanic force.

### 3.5. SR Ca^2+^ Release is Compromised in the Presence of Retinol

**Figure 5:**
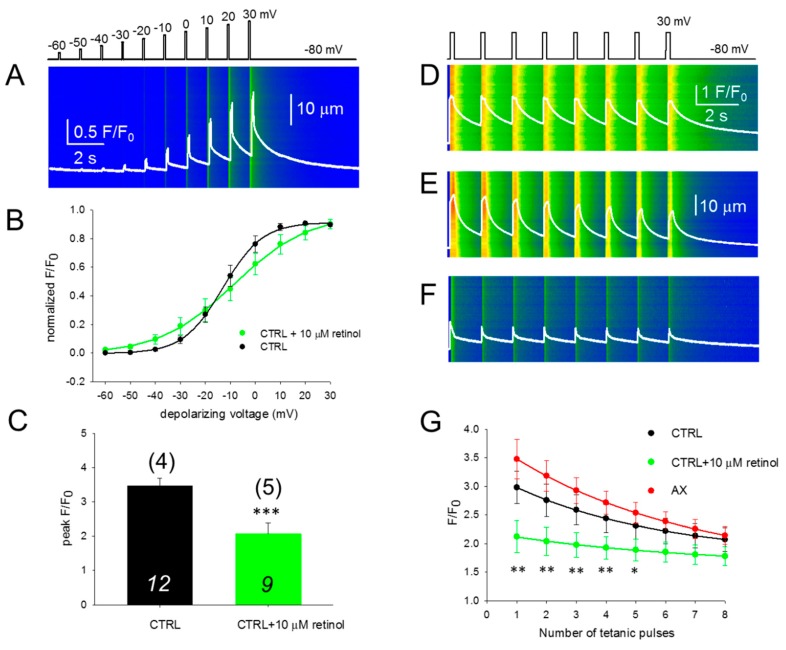
Acute retinol treatment alters the excitation-contraction coupling properties of FDB fibers. (**A**) Line-scan images of changes in intracellular calcium elicited by the same depolarization protocol as in Figure 3A,B. The white trace indicates the spatially averaged F(t)/F_0._ (**B**) Voltage dependence of the calcium transients. Values were obtained as described for Figure 3C. Continuous lines represent the best fit of the Boltzmann function to the average values with parameters of k = 7.91 and 14.76 mV, and V_50_ = –13.03 and –8.52 mV, for CTRL (*n* = 12 fibers, *N* = 4 mice) and retinol (*n* = 11 fibers, *N* = 6 mice), respectively. The control points are the same as in Figure 3C. (**C**) Normalized peak fluorescence at close to maximal depolarization (+10 mV). Values were averaged after normalization to the maximum of the individual Boltzmann fits. *** denotes significant difference (*p* < 0.001) between CTRL and retinol. (**D**–**F**) Representative line-scan images illustrating the fatigability of the FDB fibers in CTRL (**D**), AX (**E**) and retinol (**F**) treated cells. The calcium transients were elicited by a train of depolarizing pulses to +30 mV with 1.5 s delay, lasting 200 ms each. The scale corresponds to all three line-scans. (**G**) The normalized fluorescence was plotted for each pulse and single-exponential functions were fitted to the points to assess the F/F_0_ decline. The * and ** denote significant differences between AX and retinol at the given tetanus at *p* < 0.05 and *p* < 0.01, respectively. The numbers in italics indicate the number of cells studied, whereas the numbers in parenthesis denote the number of animals studied.

We performed the experiment presented in Figure 3A,B in the presence of 10 μM retinol (Figure 5A), a compound which is structurally similar to AX with different intracellular metabolism. Acute application of retinol shifted the voltage of half-maximal activation of calcium transients (Figure 5B) and altered the slope (1/k). This inhibitory effect was accompanied with a significant reduction in the peak of calcium released from the SR when the fibers were treated with retinol (Figure 5C).

### 3.6. Fatigability of Ca^2+^ Release is Decreased by AX and Increased by Retinol

Since the process of refilling regulates the steady-state SR calcium content, we were interested to see if an immediate—activation-to-activation—replenishment would also be modified by the two compounds studied here (AX and retinol) when compared to the control. To test their effects, a series of tetani mimicking maximal depolarizations with the pattern illustrated in Figure 5D were applied on CTRL (*n* = 12, Figure 5D), AX (*n* = 13, Figure 5E), and retinol treated (*n* = 8, Figure 5F) fibers from 4, 5, and 5 mice, respectively. It should be noted that the responses obtained with continuous repeated depolarizations are similar to physiological tetanic trains during muscle contraction. 

Figure 5G illustrates the evolution of F/F_0_ during repetitive stimulation in CTRL, AX, and retinol treated FDB fibers (black, red, and green traces, respectively). It is clear that during the exponential decay (using Equation (1), the best fit parameters were: *y_0_* = 1.77, 1.53 and 1.61, *a* = 1.46, 2.29 and 0.59; *b* = 0.19, 0.16 and 0.15 for control, AX and retinol treated cells, respectively) the green trace (retinol) remains well below the black trace (control) suggesting a higher propensity for depletion after acute retinol treatment. 

## 4. Discussion

Astaxanthin, an algal marine carotenoid is a potent natural antioxidant with many health benefits. With its unique polar molecular structure AX is positioned within cell membranes (preserving its structure) and circulating lipoproteins. It presents protecting effects against oxidative stress by potently quenching singlet oxygen [51]. Skeletal muscle produces ROS during contractile activity and even at rest that causes oxidative stress [1]. Redox imbalances during muscle activity can significantly contribute to the reduction of the contractile force, fatigability, and higher susceptibility to injuries. Therefore, it was proposed that dietary supplementation with antioxidants such as vitamin C, E, A, and lately carotenoids (the main precursors of fat soluble vitamin A) can decrease oxidative damage and improve performance as well as bypass muscle disorders [42,52,53]. Aoi and coworkers was first to demonstrate that AX is absorbed and transported into skeletal muscle and heart in mice [54]. However, up to date there was no information regarding AX’s effects on the ECC machinery, specifically on the function of RyR1.

In this work AstaReal A1010, an astaxanthin rich microalgal product was used which based on the product datasheet contains 5–5.6% AX, and other antioxidants such as: 0.6% d-α-tocopherol (Vitamin E) and 0.3% ascorbyl palmitate. Although one cannot exclude the possibility that these other ingredients had some effects, we are confident that in these low concentrations they did not interfere with the AX effects in this study. The applied concentration of AX was selected to match those already tested in previous studies [43]. Although the actual plasma concentration of AX was not measured either here or previously, earlier results confirmed the favorable absorption properties of algal-derived esterified AX used here [43]. It should be noted that the observed effect sizes—increase in tetanic force and reduction in mitochondrial calcium accumulation following repetitive stimulation—were modest. This however is consistent with former measurements using either other antioxidants or AX to improve muscle function or to modify body metabolism by AX [54,55,56].

### 4.1. AX and Muscle Force

The present study demonstrates that AX improves grip force in vivo (Figure 1B) and tetanic force in vitro (Figure 2C–E) which occurs in the absence of changes in the ECC machinery. Thus, alternative pathways for the improvement of muscle force generation must be considered. As a possibility we hypothesize that AX accelerated lipid utilization leading to an enhanced metabolic activity via retinoid signaling and its antioxidant effect [20]. This idea is supported by our results showing that consumption of the same amount of rodent chow (~0.2 g/day/g body weight) the AX mice gained less weight over the course of 4 weeks diet (Figure 1A). This agrees with Ikeuchi and coworkers [57] who found that AX has no effect on food intake but reduces body weight gain in obese mice possibly by increasing energy expenditure and also with Ruiz and coworkers who were first to describe the role of retinoid acid metabolism in skeletal muscle [20]. One, however, cannot exclude the possibility that AX might also alter the calcium sensitivity of the contractile filaments and thus contribute to the augmented muscle performance seen in these experiments.

### 4.2. AX and Mitochondrial Ca^2+^ Signaling

Skeletal muscle is among the most metabolically active tissues in our body hence its ATP demand is apparent. In skeletal muscle fibers mitochondria occupy 10–15% of the fiber volume and depending on the fiber type, can be localized in various proportions: (i) beneath the surface membrane; (ii) longitudinally between myofibrils; (iii) transversally at the I-band, between triads and Z-line [57]. In glycolytic fast twitch fibers (such as EDL and FDB in this study) most mitochondria are triadic, that are transversally elongated spanning across a few sarcomeres. Following muscle contraction not all calcium released from the SR leaves the muscle fibers or is re-sequestrated. Some (~10–18% of the calcium released from the SR) is taken up by the mitochondria via the mitochondrial uniporter (MCU); as calcium levels have been described to increase both in vitro and in vivo during muscle contraction and some is bound to calcium binding sites on intracellular proteins such as troponin C, parvalbumin, etc. [58,59,60,61,62,63]. Up to date, however, very few information is available on the role of coupling between mitochondria and SR in control of intracellular calcium signaling in health and disease.

Here, we provide evidence that mitochondrial calcium uptake during repetitive tetanic stimulation is reduced in the AX as compared to the control group. We observed a sustained increase in mitochondrial fluorescence (F_mito_) following stimulation that is possibly suited to promote calcium activation of ATP generation as suggested by Rossi and coworkers [62]. Our results seem to agree with the hypothesis that AX supplementation favorably alters mitochondrial, and attenuates activity dependent mitochondrial calcium unbalance [64] by possibly scavenging ROS and also by down-regulating mitochondrial calcium uptake (Figure 4).

It is possible that AX treatment changes slightly the expression level of the calcium sensitive regulator of MCU (MICU1) in skeletal muscle since we did not find differences in the mitochondrial calcium at rest or after moderate stimulation. Another possibility is that AX modifies the calcium sensibility of MICU1, since it has been reported that a splice variant of MICU1 has modified calcium binding properties [65]. Whatever the case may be this can be a protecting mechanism against mitochondrial calcium overload during heavy exercise which can initiate cell death mechanisms. On the other hand, in skeletal muscle, MICU1 modifies ATP production by directly augmenting the activity of fundamental metabolic enzymes in the mitochondria via calcium. It was shown that the lack of MICU1 did not modify mitochondrial respiration in resting muscle fibers, while it reduced the increase in oxygen consumption during calcium stimulation [66]. It is possible that AX treatment decreases the direct phosphorylation of ATP via MICU1 and shifts the energy expenditure of skeletal muscle to more fatty acid oxidation resulting in smaller body weight gain as found here. Similar results were described in a skeletal muscle-specific loss-of-function mouse model in which MICU1 was targeted [66]. Furthermore, a recent study shows that muscle-specific MICU1 ablation caused a systemic catabolic response on both liver and adipose tissue [67].

### 4.3. AX and Glucose Metabolism

As AX has been connected to metabolic changes (see above) we hypothesize that it improves muscle performance similarly to retinoic acid by possibly increasing glucose uptake and/or increasing the glycogen stores. Indeed, treatment of L6 myotubes with retinoic acid (the carboxylic acid form of retinol, Rac) enhanced insulin-stimulated glucose uptake and GLUT4 expression, reduced body weight and adiposity in lean and obese mice, increased fatty acid oxidation and irisin expression in vivo and presented a protective effect against obesity and metabolic syndrome [57,68,69,70,71]. Ruiz and coworkers found that Rac activates mTORC2 and insulin signaling in mice, and overexpression of SRP35—a small retinol dehydrogenase—improves muscle performance by increasing glucose uptake and glycogen stores [20]. Up to date, little information was available on the effect of retinol on the ECC properties in skeletal muscle. In our hands, acute treatment of FDB fibers with 10 µM retinol clearly produced a shift in the V_50_ of the voltage activated calcium transients and also significantly decreased the peak F/F_0_ (Figure 5A–C). 

**Figure 6:**
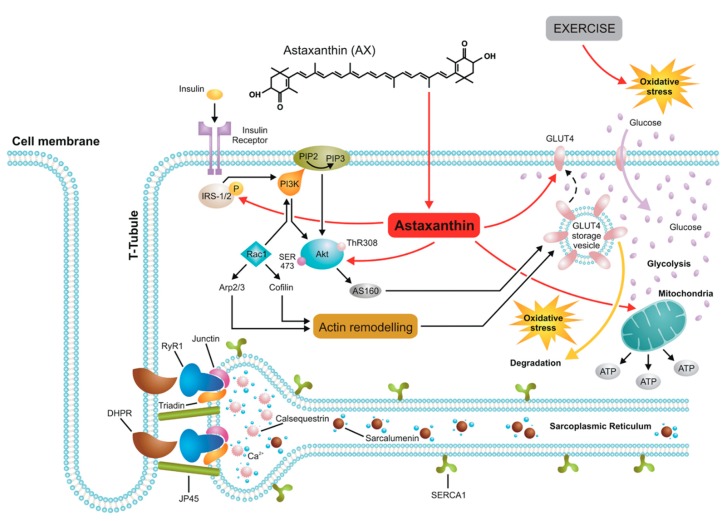
A possible model of AX actions in skeletal muscle. AX acts on the insulin receptor substrate (IRS) activating PI3K/Akt pathway which induces GLUT4 translocation into the sarcolemma resulting in enhanced glucose uptake by muscle and ultimately leading to increased glycolysis. Due to its spatial orientation—perpendicular to the plane of lipid bilayers—natural AX increases the hydrophobicity in the central core of biological membranes including the sarcolemma, sarcoplasmic reticulum (SR), and mitochondrial membranes, thus exerting its powerful antioxidant feature and protecting effects against mitochondrial calcium overload under oxidative stress conditions following exercise without altering the properties of ECC machinery.

Based on the above Figure 6 shows a schematic representation of the major cellular pathways involving AX in skeletal muscle connected to glucose homeostasis and the insulin signaling pathway. In this respect the effects of AX are similar to that of retinol. However, due to the different chemical structure—two β-ionone rings at the end of the molecule—its spatial orientation is perpendicular to the plane of lipid bilayers therefore natural AX increases the hydrophobicity in the central core of biological membranes unlike retinol which inserts into the lipids parallel to the hydrophobic tail of the lipid molecules. These features might explain the differences seen between the effects of retinol and AX on calcium signaling.

## 5. Conclusions

Our results point in the direction that AX, a generally recognized safe dietary supplement not only serves as an antioxidant but also alters mitochondrial calcium signaling without affecting ECC, could enhance glucose metabolism, and therefore contribute to improving contractile function. This opens up new treatment directions for patients suffering in metabolic diseases but further studies are needed to understand the possible underlying pathways both at cellular and at organ levels.

## Figures and Tables

**Table 1:** Force parameters from 9 control and 9 AX treated mice.

Parameters	Twitch		Tetanus	
	Control	Astaxanthin	Control	Astaxanthin
Number of muscles	9	9	9	9
Force (mN/mm^2^)	1.69 ± 0.13	1.76 ± 0.08	7.13 ± 0.49	8.67 ± 0.53 *
TTP (ms)	32.5 ± 1.4	33.1 ± 1.1	173.9 ± 5.3	169.4 ± 4.3
HRT (ms)	29.2 ± 1.0	28.8 ± 1.0	75.1 ± 6.6	81.0 ± 3.5

The * denotes significant difference between control and AX (*p* < 0.05). Abbreviations: TTP—time to peak; HRT—half relaxation time.

## References

[1] Urso M.L., Clarkson P.M. (2003). Oxidative stress, exercise, and antioxidant supplementation. Toxicology.

[2] Powers S.K., Kavazis A.N., DeRuisseau K.C. (2005). Mechanisms of disuse muscle atrophy: Role of oxidative stress. Am. J. Physiol. Regul. Integr. Comp. Physiol..

[3] Powers S.K., Nelson W.B., Hudson M.B. (2011). Exercise-induced oxidative stress in humans: Cause and consequences. Free Radic. Biol. Med..

[4] Powers S.K., Jackson M.J. (2008). Exercise-induced oxidative stress: Cellular mechanisms and impact on muscle force production. Physiol. Rev..

[5] Dröge W. (2002). Free radicals in the physiological control of cell function. Physiol. Rev..

[6] Reid M.B., Haack K.E., Franchek K.M., Valberg P.A., Kobzik L., West M.S. (1992). Reactive oxygen in skeletal muscle. I. Intracellular oxidant kinetics and fatigue in vitro. J. Appl. Physiol..

[7] O’Neill C.A., Stebbins C.L., Bonigut S., Halliwell B., Longhurst J.C. (1996). Production of hydroxyl radicals in contracting skeletal muscle of cats. J. Appl. Physiol..

[8] Aguiló A., Tauler P., Sureda A., Cases N., Tur J., Pons A. (2007). Antioxidant diet supplementation enhances aerobic performance in amateur sportsmen. J. Sports Sci..

[9] Poulsen H.E., Loft S., Vistisen K. (1996). Extreme exercise and oxidative DNA modification. J. Sports Sci..

[10] Alessio H.M., Hagerman A.E., Fulkerson B.K., Ambrose J., Rice R.E., Wiley R.L. (2000). Generation of reactive oxygen species after exhaustive aerobic and isometric exercise. Med. Sci. Sports Exerc..

[11] Reid M.B., Stokicć D.S., Koch S.M., Khawli F.A., Arturo Leis A. (1994). N-acetylcysteine inhibits muscle fatigue in humans. J. Clin. Investig..

[12] Gomez-Cabrera M., Domenech E., Viña J. (2008). Moderate exercise is an antioxidant: Upregulation of antioxidant genes by training. Free Radic. Biol. Med..

[13] Sacheck J.M., Milbury P.E., Cannon J.G., Roubenoff R., Blumberg J.B. (2003). Effect of vitamin E and eccentric exercise on selected biomarkers of oxidative stress in young and elderly men. Free Radic. Biol. Med..

[14] Venditti P., Napolitano G., Barone D., Di Meo S. (2014). Vitamin E supplementation modifies adaptive responses to training in rat skeletal muscle. Free Radic. Res..

[15] Ristow M. (2014). Unraveling the truth about antioxidants: Mitohormesis explains ROS-induced health benefits. Nat. Med..

[16] Mastaloudis A., Traber M.G., Carstensen K., Widrick J.J. (2006). Antioxidants did not prevent muscle damage in response to an ultramarathon run. Med. Sci. Sports Exerc..

[17] Finaud J., Lac G., Filaire E. (2006). Oxidative stress: Relationship with exercise and training. Sports Med..

[18] Petiz L.L., Girardi C.S., Bortolin R.C., Kunzler A., Gasparotto J., Rabelo T.K., Matté C., Moreira J.C., Gelain D.P. (2017). Vitamin A Oral Supplementation Induces Oxidative Stress and Suppresses IL-10 and HSP70 in Skeletal Muscle of Trained Rats. Nutrients.

[19] Sleeman M.W., Wortley K.E., Lai K.-M.V., Gowen L.C., Kintner J., Kline W.O., Garcia K., Stitt T.N., Yancopoulos G.D., Wiegand S.J. (2005). Absence of the lipid phosphatase SHIP2 confers resistance to dietary obesity. Nat. Med..

[20] Ruiz A., Dror E., Handschin C., Furrer R., Perez-Schindler J., Bachmann C., Treves S., Zorzato F. (2018). Over-expression of a retinol dehydrogenase (SRP35/DHRS7C) in skeletal muscle activates mTORC2, enhances glucose metabolism and muscle performance. Sci. Rep..

[21] Nishida Y., Yamashita E., Miki W. (2007). Quenching Activities of Common Hydrophilic and Lipophilic Antioxidants against Singlet Oxygen Using Chemiluminescence Detection System. Carotenoid Sci..

[22] Sawaki K., Yoshigi H., Aoki K., Koikawa N., Azumane A., Kaneko K., Yamaguchi M. (2002). Sports Performance benefits from taking Natural Astaxanthin characterized by visual acuity and muscle fatigue improvements in humans. Therap. Med..

[23] Fukamauchi M. (2007). Food functionality of astaxathin-10: Synergistic effects of astaxanthin-10 intake and aerobic exercise. Food Style 21.

[24] Wolf A.M., Asoh S., Hiranuma H., Ohsawa I., Iio K., Satou A., Ishikura M., Ohta S. (2010). Astaxanthin protects mitochondrial redox state and functional integrity against oxidative stress. J. Nutr. Biochem..

[25] Rodriguez-Amaya D.B. (2010). Quantitative Analysis, in Vitro Assessment of Bioavailability and Antioxidant Activity of Food Carotenoids—A Review. J. Food Compos. Anal..

[26] Hussein G., Sankawa U., Goto H., Matsumoto K., Watanabe H. (2006). Astaxanthin, a carotenoid with potential in human health and nutrition. J. Nat. Prod..

[27] Ambati R.R., Phang S.M., Ravi S., Aswathanarayana R.G. (2014). Astaxanthin: Sources, extraction, stability, biological activities and its commercial applications—A review. Mar. Drugs.

[28] Sztretye M., Dienes B., Gönczi M., Czirják T., Csernoch L., Dux L., Szentesi P., Keller-Pintér A. (2019). Astaxanthin: A Potential Mitochondrial-Targeted Antioxidant Treatment in Diseases and with Aging. Oxid. Med. Cell. Longev..

[29] Zhang Z.W., Xu X.C., Liu T., Yuan S. (2016). Mitochondrion-permeable antioxidants to treat ROS-burst-mediated acute diseases. Oxid. Med. Cell. Longev..

[30] Kuroki T., Ikeda S., Okada T., Maoka T., Kitamura A., Sugimoto M., Kume S. (2013). Astaxanthin ameliorates heat stress-induced impairment of blastocyst development in vitro: Astaxanthin colocalization with and action on mitochondria. J. Assist. Reprod. Genet..

[31] Tanabe T., Beam K.G., Numa S. (1990). Regions of the skeletal muscle dihydropyridine receptor critical for excitation–contraction coupling. Nature.

[32] MacLennan D.H. (2000). Ca2+ signalling and muscle disease. Eur. J. Biochem..

[33] Hovnanian A. (2007). SERCA pumps and human diseases. Subcell Biochem..

[34] Rossi A.E., Dirksen R.T. (2006). Sarcoplasmic reticulum: The dynamic calcium governor of muscle. Muscle Nerve.

[35] Gunter T.E., Buntinas L., Sparagna G., Eliseev R., Gunter K. (2000). Mitochondrial calcium transport: Mechanisms and functions. Cell Calcium.

[36] Gunter T.E., Yule D.I., Gunter K.K., Eliseev R.I., Salter J.D. (2004). Calcium and mitochondria. FEBS Lett..

[37] Lawler J.M., Hu Z., Barnes W.S. (1998). Effect of reactive oxygen species on K+ contractures in the rat diaphragm. J. Appl. Physiol..

[38] Dulhunty A.F., Zhu P.H. (1993). Do independent processes control the activation and inactivation of potassium contracture tension in rat skeletal muscle?. J. Membr. Biol..

[39] Liu G., Abramson J.J., Zable A.C., Pessah I.N. (1994). Direct evidence for the existence and functional role of hyperreactive sulfhydryls on the ryanodine receptor-triadin complex selectively labelled by the coumarin maleimide 7-diethylamino-3-(4′-maleimidylphenyl)-4-methylcoumarin. Mol. Pharmacol..

[40] Aghdasi B., Zhang J.Z., Wu Y., Reid M.B., Hamilton S.L. (1997). Multiple classes of sulfhydryls modulate the skeletal muscle Ca2+ release channel. J. Biol. Chem..

[41] Andersson B.C., Betzenhauser M.J., Reiken S., Meli A.C., Umanskaya A., Xie W., Shiomi T., Zalk R., Lacampagne A., Marks A.R. (2011). Ryanodine Receptor Oxidation Causes Intracellular Calcium Leak and Muscle Weakness in Aging. Cell Metab..

[42] Aoi W., Naito Y., Sakuma K., Kuchide M., Tokuda H., Maoka T., Toyokuni S., Oka S., Yasuhara M., Yoshikawa T. (2003). Astaxanthin limits exercise-induced skeletal and cardiac muscle damage in mice. Antioxid. Redox Signal..

[43] Aoi W., Maoka T., Abe R., Fujishita M., Tominaga K. (2018). Comparison of the effect of non-esterified and esterified astaxanthins on endurance performance in mice. J. Clin. Biochem. Nutr..

[44] Visioli F., Artaria C. (2017). Astaxanthin in cardiovascular health and disease: Mechanisms of action, therapeutic merits, and knowledge gaps. Food Funct..

[45] Bodnár D., Geyer N., Ruzsnavszky O., Oláh T., Hegyi B., Sztretye M., Fodor J., Dienes B., Balogh Á., Papp Z. (2014). Hypermuscular mice with mutation in the myostatin gene display altered calcium signaling. J. Physiol..

[46] Bodnár D., Ruzsnavszky O., Oláh T., Dienes B., Balatoni I., Ungvári É., Benkő I., Babka B., Prokisch J., Csernoch L. (2016). Dietary selenium augments sarcoplasmic calcium release and mechanical performance in mice. Nutr. Metab..

[47] Szentesi P., Jacquemond V., Kovács L., Csernoch L. (1997). Intramembrane charge movement and sarcoplasmic calcium release in enzymatically isolated mammalian skeletal muscle fibres. J. Physiol..

[48] Fodor J., Gonczi M., Sztretye M., Dienes B., Olah T., Szabo L., Csoma E., Szentesi P., Szigeti G.P., Marty I. (2008). Altered expression of triadin 95 causes parallel changes in localized Ca2+ release events and global Ca2+ signals in skeletal muscle cells in culture. J. Physiol..

[49] Sztretye M., Geyer N., Vincze J., Al-Gaadi D., Oláh T., Szentesi P., Kis G., Balatoni I., Csernoch L., Dienes B. (2017). Store-operated calcium entry is important for maintaining sarcoplasmic calcium content and release in mammalian skeletal muscle fibers. Biophys. J..

[50] Ainbinder A., Boncompagni S., Protasi F., Dirksen R.T. (2015). Role of Mitofusin-2 in mitochondrial localization and calcium uptake in skeletal muscle. Cell Calcium.

[51] Fassett R.G., Coombes J.S. (2012). Astaxanthin in Cardiovascular Health and Disease. Molecules.

[52] Evans W.J. (2000). Vitamin E, vitamin C, and exercise. Am. J. Clin. Nutr..

[53] Aoi W., Naito Y., Takanami Y., Ishii T., Kawai Y., Akagiri S., Kato Y., Osawa T., Yoshikawa T. (2008). Astaxanthin improves muscle lipid metabolism in exercise via inhibitory effect of oxidative CPT I modification. Biochem. Biophys. Res. Commun..

[54] Mao G., Kraus G.A., Kim I., Spurlock M.E., Bailey T.B., Beitz D.C. (2011). Effect of a mitochondria-targeted vitamin E derivative on mitochondrial alteration and systemic oxidative stress in mice. Br. J. Nutr..

[55] Kanazashi M., Tanaka M., Nakanishi R., Maeshige N., Fujino H. (2019). Effects of astaxanthin supplementation and electrical stimulation on muscle atrophy and decreased oxidative capacity in soleus muscle during hindlimb unloading in rats. J. Physiol. Sci..

[56] Ikeuchi M., Koyama T., Takahashi J., Yazawa K. (2007). Effects of Astaxanthin in Obese Mice Fed a High-Fat Diet. Biosci. Biotechnol. Biochem..

[57] Boncompagni S., Rossi A.E., Micaroni M., Beznoussenko G.V., Polishchuk R.S., Dirksen R.T., Protasi F. (2009). Mitochondria are linked to calcium stores in striated muscle by developmentally regulated tethering structures. Mol. Biol. Cell.

[58] Pan X., Liu J., Nguyen T., Liu C., Sun J., Teng Y., Fergusson M.M., Rovira I.I., Allen M., Springer D.A. (2013). The physiological role of mitochondrial calcium revealed by mice lacking the mitochondrial calcium uniporter. Nat. Cell Biol..

[59] De Stefani D., Raffaello A., Teardo E., Szabò I., Rizzuto R. (2011). A forty-kilodalton protein of the inner membrane is the mitochondrial calcium uniporter. Nature.

[60] Paillard M., Csordás G., Szanda G., Golenár T., Debattisti V., Bartok A., Wang N., Moffat C., Seifert E.L., Spät A. (2017). Tissue-Specific Mitochondrial Decoding of Cytoplasmic Ca2+ Signals Is Controlled by the Stoichiometry of MICU1/2 and MCU. Cell Rep..

[61] Yi J., Ma C., Li Y., Weisleder N., Rios E., Ma J., Zhou J. (2011). Mitochondrial calcium uptake regulates rapid calcium transients in skeletal muscle during excitation–contraction (E-C) coupling. J. Biol. Chem..

[62] Rossi A.E., Boncompagni S., Wei L., Protasi F., Dirksen R.T. (2011). Differential impact of mitochondrial positioning on mitochondrial Ca2+ uptake and Ca2+ spark suppression in skeletal muscle. Am. J. Physiol. Cell Physiol..

[63] Rudolf R., Mongillo M., Magalhães P.J., Pozzan T. (2004). In vivo monitoring of Ca2+ uptake into mitochondria of mouse skeletal muscle during contraction. J. Cell Biol..

[64] Polotow T.G., Vardaris C.V., Mihaliuc A.R., Gonçalves M.S., Pereira B., Ganini D., Barros M.P. (2014). Astaxanthin supplementation delays physical exhaustion and prevents redox imbalances in plasma and soleus muscles of Wistar rats. Nutrients.

[65] Vecellio Reane D., Vallese F., Checchetto V., Acquasaliente L., Butera G., De Filippis V., Szabò I., Zanotti G., Rizzuto R., Raffaello A. (2016). A MICU1 Splice Variant Confers High Sensitivity to the Mitochondrial Ca2+ Uptake Machinery of Skeletal Muscle. Mol. Cell.

[66] Kwong J.Q., Huo J., Bround M.J., Boyer J.G., Schwanekamp J.A., Ghazal N., Maxwell J.T., Jang Y.C., Khuchua Z., Shi K. (2018). The mitochondrial calcium uniporter underlies metabolic fuel preference in skeletal muscle. JCI Insight.

[67] Gherardi G., Nogara L., Ciciliot S., Fadini G.P., Blaauw B., Braghetta P., Bonaldo P., De Stefani D., Rizzuto R., Mammucari C. (2019). Loss of mitochondrial calcium uniporter rewires skeletal muscle metabolism and substrate preference. Cell Death Differ..

[68] Sleeman M.W., Zhou H., Rogers S., Ng K.W., Best J.D. (1995). Retinoic acid stimulates glucose transporter expression in L6 muscle cells. Mol. Cell Endocrinol..

[69] Bonet M.L., Ribot J., Palou A. (2012). Lipid metabolism in mammalian tissues and its control by retinoic acid. Biochim. Biophys. Acta.

[70] Arunkumar E., Bhuvaneswari S., Anuradha C.V. (2012). An intervention study in obese mice with astaxanthin, a marine carotenoid--effects on insulin signaling and pro-inflammatory cytokines. Food Funct..

[71] Amengual J., García-Carrizo F.J., Arreguín A., Mušinović H., Granados N., Palou A., Bonet M.L., Ribot J. (2018). Retinoic Acid Increases Fatty Acid Oxidation and Irisin Expression in Skeletal Muscle Cells and Impacts Irisin In Vivo. Cell Physiol. Biochem..

